# Predicting response speed and age from task-evoked effective connectivity

**DOI:** 10.1162/netn_a_00447

**Published:** 2025-04-30

**Authors:** Shufei Zhang, Kyesam Jung, Robert Langner, Esther Florin, Simon B. Eickhoff, Oleksandr V. Popovych

**Affiliations:** Institute of Neuroscience and Medicine, Brain and Behaviour (INM-7), Research Centre Jülich, Jülich, Germany; Institute for Systems Neuroscience, Medical Faculty, Heinrich-Heine University Düsseldorf, Düsseldorf, Germany; Institute of Clinical Neuroscience and Medical Psychology, Medical Faculty, Heinrich-Heine University Düsseldorf, Dsseldorf, Germany

**Keywords:** Task fMRI, Dynamic causal modeling, Analytic flexibility, Machine learning, Brain-based prediction, Stimulus-response compatibility, Functional connectivity

## Abstract

Recent neuroimaging studies demonstrated that task-evoked functional connectivity (FC) may better predict individual traits than resting-state FC. However, the prediction properties of task-evoked effective connectivity (EC) remain unexplored. We investigated this by predicting individual reaction time (RT) performance in the stimulus-response compatibility task and age, using intrinsic EC (I-EC; calculated at baseline) and task-modulated EC (M-EC; induced by experimental conditions) with dynamic causal modeling (DCM) across various data processing conditions, including different general linear model (GLM) designs, Bayesian model reduction, and different cross-validation schemes and prediction models. We report evident differences in predicting RT and age between I-EC and M-EC, as well as between event-related and block-based GLM and DCM designs. M-EC outperformed both I-EC and task-evoked FC in RT prediction, while all types of connectivity performed similarly for age. Event-related GLM and DCM designs performed better than block-based designs. Our findings suggest that task-evoked I-EC and M-EC may capture different phenotypic attributes, with performance influenced by data processing and modeling choices, particularly the GLM-DCM design. This evaluation of methods for behavior prediction from brain EC may contribute to a meta-scientific understanding of how data processing and modeling frameworks influence neuroimaging-based predictions, offering insights for improving their robustness and efficacy.

## Author Summary

We investigated how brain task-evoked effective connectivity (EC) can predict individual differences in behavior and age. We examined two types of EC: intrinsic EC (calculated at baseline) and task-modulated EC (induced by experimental conditions) calculated by dynamic causal modeling across various data processing conditions. We found that the task-modulated EC outperformed intrinsic EC in predicting reaction time measured during a stimulus-response task, while both EC types performed similarly in age prediction. Our findings may suggest that different EC types could capture distinct phenotypic traits, with performance influenced by data processing and modeling choices. This evaluation may further promote the application of model-based approaches to behavior prediction from brain connectivity and enhance our understanding of the impact of data processing on the prediction results.

## INTRODUCTION

Linking individual differences in behavior with individual brain properties is one of the main goals of cognitive neuroscience (Biswal et al., 2010; Huth et al., 2016). Functional magnetic resonance imaging (fMRI) has been instrumental in predicting individual behavior and phenotypes by modeling brain activation and connectivity patterns (Biswal et al., 2010; Fox et al., 2007; van den Heuvel & Pol, 2010). Functional connectivity (FC), the correlation between blood-oxygen-level-dependent (BOLD) signal fluctuations of two brain regions, reveals task-evoked and resting-state coactivation patterns (Biswal et al., 2010; Cole et al., 2014). Although resting-state FC has been widely studied for predicting behaviors and phenotypes (Dosenbach et al., 2010; Heckner et al., 2023; Sripada et al., 2020), accumulating evidence indicates that task-evoked FC may better capture intersubject differences than resting-state FC (Finn & Bandettini, 2021; Finn et al., 2017; Rosenberg et al., 2016), with studies showing improved predictions for reading skills (Jiang et al., 2020), fluid intelligence (Greene et al., 2018), and general cognitive ability (Zhao et al., 2023)—but see Kraljević et al. (2024) for contradictory evidence.

The correlational nature of FC is agnostic to the causal interdependencies between brain regions. These causal aspects of interregional coupling may however be especially informative about individual differences in behavior when studying connectivity patterns during task states. Recent achievements in generative embedding methods have provided powerful frameworks for capturing causal interdependence. Among a few generative embedding methods (Rajapakse & Zhou, 2007; Roebroeck et al., 2005; Schlösser et al., 2003), dynamic causal modeling (DCM) (Friston et al., 2003) offers a biologically meaningful approach to estimating brain effective connectivity (EC) from task-evoked fMRI. Within this framework, EC is inferred from DCM in order to estimate the directional influence of a given brain region on another (Friston et al., 2003) and is intended to provide deeper, mechanistic insights by explicitly modeling such causal influences (Friston, 2011). This characteristic may contribute more discriminative features to EC to better identify individual “fingerprints” (Geng et al., 2018). In DCM, intrinsic EC (I-EC) corresponds to the A matrix in the DCM equation (see the Methods section), representing baseline connectivity in the absence of task modulation, while task-modulated EC (M-EC) corresponds to the B matrix, capturing changes in connectivity driven by specific experimental conditions (Zeidman, Jafarian, Corbin, et al., 2019). These components thus reflect brain connectivity at baseline and in task-modulated states, respectively.

Building on this, we aimed to explore whether I-EC and M-EC can display such differential roles in prediction. Both of them have been investigated with respect to a range of human cognitive functions such as working memory, finger tapping, response conflict resolution, and reading as well as aging at the level of group-average effects (Boudrias et al., 2012; Cieslik et al., 2011; Jung et al., 2018; Kahan et al., 2019; Loehrer et al., 2016; Morken et al., 2017; Volz et al., 2015). With regard to interindividual differences, recent studies have begun to utilize EC modeling approaches to predict individual behaviors and phenotypes including age differences and task performance either from I-EC or M-EC (Beck et al., 2021; Brodersen et al., 2011; Diersch et al., 2021; Tsvetanov et al., 2016). However, in contrast to extensive comparisons of predictive performance between different types of FC (Greene et al., 2018), prediction performance based on task-evoked EC features and, in particular, the difference between features derived from I-EC versus M-EC components remains unexplored.

We addressed this by using task-evoked EC modeled via DCM for the prediction of (a) individual average reaction time (RT) of incongruent conditions during a spatial stimulus-response compatibility (SRC) task (Fitts & Deininger, 1954; Langner et al., 2015) and (b) age as two different (behavioral and demographic) individual characteristics. The spatial SRC task is designed to probe cognitive action control during response conflict processing, associated with an increase of RT in the incongruent condition, relative to the congruent one (Langner et al., 2015), and this incongruency effect was found to be further enhanced in advanced age (Proctor et al., 2005). These characteristics have motivated our endeavor to predict individual age and cognitive action control, as reflected in the incompatibility effects measured by RT. Predicting the distinct characteristics such as age and RT is also important for understanding changes in brain neural dynamics during the SRC task by contrasting the prediction performances for these different behavioral/phenotypic scores.

In this study, we compared the prediction results for individual RT and age using I-EC and M-EC components derived from fMRI data obtained during SRC task performance. Although the I-EC component inferred from task-evoked functional data is not entirely independent of the task-related contexts, it is still considered to reflect brain intrinsic connectivity dynamics by mathematically removing task-relevant variance (Marreiros et al., 2008; Zeidman, Jafarian, Corbin, et al., 2019). This makes it a reasonable candidate for comparison with M-EC components in predictions. Our approach included the following steps: (a) We extracted nodes of the SRC network and estimated I-EC and M-EC for each participant. (b) We calculated the prediction accuracy of I-EC and M-EC for RT and age predictions using 5-fold cross-validation (CV) and conducted sensitivity analyses to examine the impact of various factors. These factors included different general linear model (GLM) and DCM designs (Huettel, 2012; Logothetis, 2008), multiple CV schemes consisting of a 10-fold CV and leave-one-out CV (LOOCV), as well as the Bayesian model reduction (BMR) approach (Friston et al., 2016). (c) We compared the prediction accuracy of the task-evoked EC and FC. Since EC patterns may depend on the sample size (Silchenko et al., 2023), we applied our prediction analyses to a relatively large group of participants (*n* > 200). We in particular show that the type of GLM/DCM design (event-related vs. block-based) and EC modality (intrinsic at baseline vs. task-modulated) have a crucial influence on prediction performance, while the other conditions considered have a rather weak impact. Furthermore, task-evoked EC was found to outperform FC in RT prediction, but not in age prediction.

## METHODS

This study investigated the application of task-evoked EC calculated by the DCM approach to the prediction of behavioral data of individual subjects by using a machine-learning approach. We considered the spatial SRC task (Fitts & Deininger, 1954), calculated the task-evoked EC of the respective task-evoked SRC brain network, and used linear regression with the least absolute shrinkage and selection operator (LASSO) regularization (Tibshirani, 1996) to predict RT and age of individual subjects. This was replicated using ridge regression and compared with prediction results based on task-evoked FC features. Furthermore, we considered typical points of analytical flexibility and investigated the impact of data processing, EC calculation, feature extraction, and prediction procedure as explained in detail below.

### Experimental Protocol

The present study used fMRI data recorded while participants performed a spatial SRC task (Fitts & Deininger, 1954; Langner et al., 2015). This two-choice reaction task consisted of 24 blocks of trials with response requirements that were either spatially compatible (“Pro”) or incompatible (“Anti”) with a visual stimulus (Supporting Information Figure S1a). In particular, participants were instructed to respond to lateralized visual stimuli by accurately and rapidly pressing an ipsilateral (Pro) or contralateral button (Anti), respectively. Before each block, a 2-s instruction was presented to indicate the upcoming condition (Pro or Anti), and each block contained 13 to 16 trials, in which the stimulus was presented for 0.2 s at the beginning of each trial on either the left or right side of the screen with equal probability (50%) for each side. The intervals between trial onsets randomly varied from 2 to 4.5 s according to a uniform distribution, and rest periods between blocks ranging from 15 to 19 s were also randomly jittered according to a uniform distribution. Each task condition (Pro and Anti) was repeated 12 times, with conditions (blocks) and stimuli within each condition being presented in a pseudorandomized order. Trials with responses being too fast or too slow (RT < 150 ms or RT > 1,500 ms) were excluded, and individual average RT of incongruent (Anti) conditions was extracted as the prediction target.

### Participants

Our study included an initial sample of 271 subjects (148 males, 123 females, 18–85 years old, *M*_age_ = 52.3 ± 16.6 years) recruited from the subject pool of the 1000BRAINS project (Caspers et al., 2014), which was conducted at the Research Centre Jülich. Before entering the study, the written informed consent of each subject was acquired. The study protocol was approved by the ethics committee of the University Duisburg-Essen (reference number: 11-4678) and performed in accordance with the declaration of Helsinki.

### MRI Data Acquisition

Details about MRI data included in the 1000BRAINS project can be found elsewhere (Caspers et al., 2014). Structural MRI scans were obtained using an anatomical 3D T1w MPRAGE sequence (Magnetization Prepared Rapid Gradient Echo) with the following parameters: repetition time (TR) = 2.0 s, echo time (TE) = 3.03 ms, flip angle = 9^o^, 176 sagittal slices, field of view = 256 mm, voxel resolution = 1 × 1 × 1 mm^3^.

The task fMRI scans were obtained by a gradient-echo echo-planar imaging sequence with the following parameters: TR = 2.03 s, TE = 30 ms, flip angle = 80^o^, field of view = 200 mm, 33 axial slices (ascending), slice thickness = 3.3 mm, interslice gap = 0.66 mm, voxel resolution = 3.1 × 3.1 × 3.3 mm^3^, acquisition time = 27 min and 10 s.

### Data Processing

**Figure 1.**
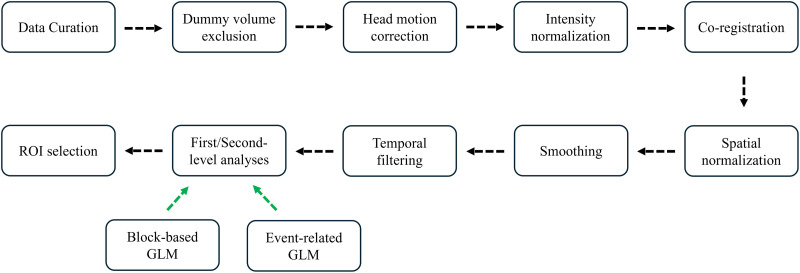
Flow chart for the stimulus-response compatibility task-evoked network node selection. The dashed arrows indicate the processing flow. Abbreviations: ROI, regions of interest; GLM, general linear model.

**Figure 2.**
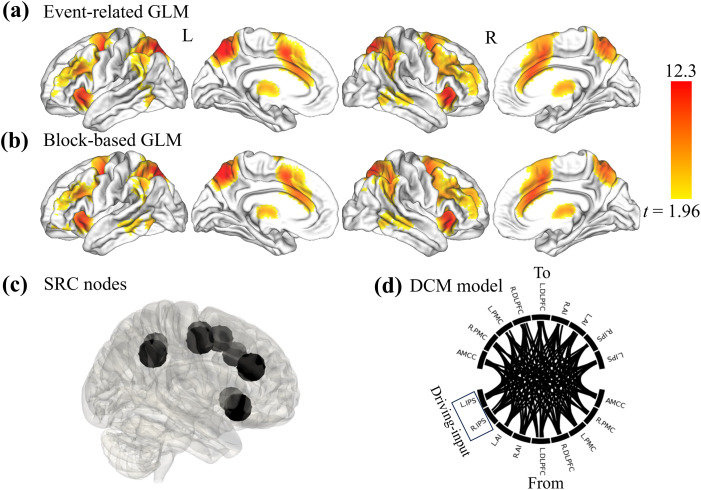
The general DCM workflow. (a, b) Second-level fMRI statistics computed from the incompatible (Anti) > compatible (Pro) activation contrasts. Maps illustrate the *t* values (scaling given in the color bar) of the *t* tests reflecting the statistically significant voxels across all subjects with the threshold-free cluster-enhancement (TFCE) and family-wise error rate (FWE) methods (*p*_TFCE+FWE_ < 0.05). (c) Example of SRC nodes extracted from the maps illustrated in plots (a, b). (d) Schematic illustration of the full-connection DCM model that was implemented in the analysis of effective connectivity (EC). Abbreviations: L/R, left/right; DLPFC, dorsolateral prefrontal cortex; PMC, premotor cortex; IPS, intraparietal sulcus; AI, anterior insula; AMCC, anterior midcingulate cortex. Events, experimental designs with all trials; Block, experimental designs modeled by blocks; TFCE, threshold-free cluster enhancement; FWE, family-wise error.

In our previous study (Zhang et al., 2024), several data processing and EC calculation conditions were considered with a particular focus on the type of GLM design. The present study aimed to investigate the prediction performance of I-EC and M-EC by considering two different GLM designs (event-related vs. block-based). Briefly, the preprocessing steps (Figure 1) included dummy-volume exclusion, head-motion correction, intensity normalization, co-registration, spatial normalization, smoothing (8-mm Gaussian kernel), covariance regression (24 head-motion parameters, and regressors of white-matter, cerebrospinal-fluid, and global signals), and high-pass temporal filtering (128 s). The individual BOLD signals were modeled through the utilization of two distinct GLM designs: event-related and block-based designs (Supporting Information Figures S1b and S1c). In the event-related design, a step function with a specific onset time of an individual stimulus and a fixed “on” duration of 0.2 s were used for each stimulus. The block-based design incorporated each block’s starting time and full duration as the onset time and duration, respectively. Then, the second-level GLM analyses were used to derive activation maps specifically related to stimulus-response incompatibility (i.e., Anti > Pro condition contrasts; Figures 2a and 2b) for block-based and event-related designs using FSL (Version 6.0) (https://fsl.fmrib.ox.ac.uk/fsl/fslwiki). Afterward, nine regions of interest (ROIs;10 mm radius) of brain activation were defined based on the observed activation peaks leading to the group-level SRC-related brain network (Figure 2c): anterior midcingulate cortex (AMCC), bilateral intraparietal sulcus (IPS), premotor cortex (PMC), dorsolateral prefrontal cortex (DLPFC), and anterior insula (AI). The selection of ROIs agrees with previous literature on SRC tasks, where both the aging and incompatibility effects on them have been reported (Cieslik et al., 2010; Langner et al., 2015). These ROIs were then overlaid with the first-level GLM-derived activation map related to performing the Anti condition of each subject thresholded at *p* < 0.05 and to search for subject-specific activation peaks within the group-level ROIs and dilate these peaks into spheres with a 4-mm radius. Finally, the BOLD time series for all nine individual ROIs (first eigenvariate of all significant voxels within a sphere) were estimated for each subject, which was then modeled by DCM for the full-connection model. For the event-related and block-based GLM and DCM designs, 210 and 213 participants, respectively, were qualified for the extraction of individual BOLD signals of the SRC network, because not all individual ROIs contained significant voxels for some subjects. More details about fMRI data processing as well as extraction of the task-evoked BOLD signals of the SRC network of individual subjects for DCM calculation can be found in Zhang et al. (2024) and in the Supporting Information.

### DCM Specification and Analysis

The DCM analysis (Friston et al., 2003) was conducted based on the following model:

$$\frac{dz}{dt}=\left(A+\sum_{k}B^{\left(k\right)}u_{k}\left(t\right)\right)z+Cu\left(t\right).$$

Here, *z* represents the neural states of network ROIs across time points, the matrices *A* and *B* stand for parameters of intrinsic and task-modulated connectivity, respectively, and *u*_*k*_(*t*) encodes the timing of the experimental condition *k*. Matrix *C* represents the influence of all external experimental inputs (stimulation) *u*(*t*) on the neural dynamics of the considered ROIs. The observed BOLD response *y*(*t*) is modeled by the observation equation:

$$y\left(t\right)=h\left(z\left(t\right)+\epsilon \left(t\right)\right)$$

where *h*(·) represents the hemodynamic response function and *ϵ*(*t*) indicates the noise. The model was used to infer the coupling parameters of the above matrices such that the simulated BOLD signals best explained the variance of the empirical BOLD signals.

Several DCM parameters are therefore defined as follows: (a) *Driving input* (matrix C) that defines external visual input to the network. Given that IPS nodes were identified as pivotal hubs for sensorimotor integration during visually guided actions (Anderson et al., 2014), we designated the bilateral IPS nodes as the primary driving-input nodes, responsible for receiving external (visual) input in our DCM model (Figure 2d). (b) I-EC as given by the connectivity matrix A denotes the unmodulated EC that exists among the network nodes at baseline, that is, in the absence of any experimental task and its modulations. Although I-EC was inferred from our SRC task-related fMRI data, it captures the intrinsic coupling between brain network nodes by mathematically removing task-related variance (Marreiros et al., 2008; Zeidman, Jafarian, Corbin, et al., 2019). (c) *Modulatory EC* (M-EC) as given by the connectivity matrix B that reflects the modulation of EC connections in response to a certain task condition. The interpretation of the connectivity matrices A and B as representing I-EC and M-EC, respectively, depends on how the experimental input *u*_*j*_ is handled. In this study, we considered *u*_*j*_ to vary between 0 and 1, corresponding to the task conditions being off (0) or on (1), instead of mean-centering *u*_*j*_. When *u*_*j*_ is not mean-centered, as in our case, matrix A reflects the EC parameters of the unmodelled baseline, and matrix B represents the EC parameters modulated by task conditions (Zeidman, Jafarian, Corbin, et al., 2019). Otherwise, if *u*_*j*_ is mean-centered, matrices A and B will represent the averaged EC across all task conditions (on and off) and EC deviations from this overall mean, respectively (Zeidman, Jafarian, Corbin, et al., 2019).

In this study, the timing of the stimulus presentation in the Anti task blocks was selected as the timing of the modulatory input, and the respective contrast was modeled by DCM, which may represent the connections modulated by individual incompatible responses of the SRC task. In comparison with the Pro condition, the Anti condition is expected to pose higher cognitive demands due to the requirement to resolve spatial incompatibility-induced response conflicts. Consequently, individual performance and estimated EC of the Anti condition can be considered robust indicators reflecting the individual capability to execute and control a complex cognitive action. This way, the DCM design corresponded to that of the GLM used for analyzing the task fMRI data, that is, event-related and block-based designs were simultaneously used for both GLM and DCM. For the event-related design, driving stimuli of all events and modulatory stimuli of the Anti condition were encoded by separate trials, whereas, for the block-based design, driving stimuli of all events and modulatory stimuli of the Anti condition were encoded by blocks. The detailed information can be found in Zhang et al. (2024).

A full-connection model was implemented for estimating I-EC and M-EC (Figure 2d). After individual I-EC and M-EC were inferred, subjects with a low fraction (< 10%) of variance explained by DCM were excluded, after which 208 and 205 participants for event-related and block-based designs, respectively, remained for subsequent analyses. Parametric Empirical Bayes (PEB) is a hierarchical modeling approach that can in particular be used to relate brain EC to behaviorally relevant characteristics, allowing for inferences on intersubject differences at the second level (Zeidman, Jafarian, Seghier, et al., 2019). With PEB, one can also estimate brain EC patterns that best explain the average connection across subjects as well as the interindividual EC differences modulated by individual RT or age by utilizing a GLM design matrix. In this context, the design matrix consists of two columns, where the first column contains all ones for the group-mean effects, while the second column includes mean-centered RT or age scores to estimate RT- or age-modulated effects. Such a design matrix enables assessing how individual differences in EC are associated with other individual (e.g., behavioral) characteristics.

As mentioned above, the estimated EC parameters were used as features to predict both individual age and RT averaged over all trials of the incongruent SRC task condition. To keep the number of covariates at a minimum (https://en.wikibooks.org/wiki/SPM/Parametric_Empirical_Bayes_(PEB)), we adopted a two-column design matrix of PEB analysis, which only contained all ones and mean-centered RT or age separately. Additionally, a threshold of posterior probability (PP > 95%) was used to extract I-EC and M-EC parameters that were strongly modulated by RT or age.

### Machine-Learning Prediction Analysis

**Figure 3.**
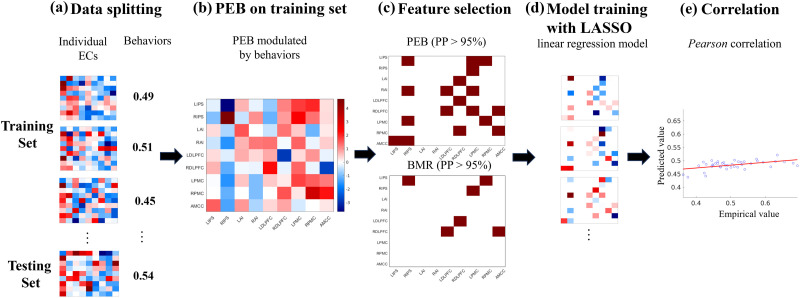
The workflow for prediction of individual behavioral characteristics based on task-evoked EC and PEB results as outlined by Steps (a)-(e). (a) The subjects and their corresponding behavioral characteristics (RT and age) were randomly split into k groups according to the *k*-fold cross-validation (CV) approach and then united into training and testing sets within CV loops. (b) Individual EC and behavior scores from the training set were analyzed by a PEB analysis to estimate behavior-modulated EC at the group level. (c) EC features are masked based on a high posterior probability (PP > 95%) obtained from PEB or Bayesian model reduction (BMR) analysis. (d) A linear regression model with LASSO regularization was trained on the training set and then applied to predict the behavioral/phenotypic scores of the unseen testing set. (e) Pearson correlation was used to evaluate the similarity between the empirical and predicted values of the testing sets and to indicate the prediction accuracy of the model’s performance by averaging the correlation across all testing sets of the CV loop. The procedures (a)–(e) were repeated for several random splits of subjects into k groups to obtain a distribution of the prediction accuracies.

The main goal of this study was to compare the prediction accuracy of task-evoked EC of the SRC task network (defined via block-based vs. event-related GLM and DCM designs) with respect to age as well as individual performance level as given by RT. I-EC and M-EC matrices were selected as candidate features, and individual age and RT of the incompatible task condition were chosen as target characteristics to be predicted. An approach to feature selection by masking the features at the group level is widely used in prediction analyses, see, for example, the connectome-based predictive modeling (CPM) approach (Shen et al., 2017). In our study, we considered EC and thus used PEB for the feature selection, since the latter was recommended in the literature for group-level EC analyses (Zeidman, Jafarian, Seghier, et al., 2019). We applied a linear regression model with the LASSO regularization (Tibshirani, 1996) to predict individual differences (RT and age) using the *k*-fold CV scheme. LASSO was chosen for its ability to reduce overfitting, especially in high-dimensional data. By penalizing features with less importance, LASSO ensures that the prediction model focuses on predictive EC parameters, which improves the model’s accuracy and generalizability. In such a way, we randomly split the subject sample into five groups, where four groups were united into a set for model training, whereas the fifth group was kept for model testing and prediction of individual target scores (Figure 3a).

To select EC parameters to be used as features for prediction, a group-level PEB (Zeidman, Jafarian, Seghier, et al., 2019) framework of DCM was employed. During the PEB analysis on the training sets, a PEB design matrix with two columns was used, as mentioned above, to extract the connectivity patterns of strongly evident (with PP > 95%) group-mean EC parameters as well as EC parameters modulated by mean-centered individual age or RT (Figure 3b). These modulated EC parameters at the second-level (PP > 95%) were selected as a mask to extract subject-level EC parameters without any refitting procedures for individuals, which were then used as features to train the prediction model on the training sets and to predict individual RT or age for the testing sets of unseen subjects (Figures 3c and 3d). One prediction round included testing all five subject groups of the 5-fold CV split by selecting them one after another as a testing set (and the respective remainder of the sample as a training set), where all test subjects obtained predicted scores that were compared (correlated) with the empirical ones across subjects (Figure 3e). This procedure was repeated 100 times with different random splits of the sample into five groups, and we obtained a distribution of 100 values of prediction accuracy (i.e., Pearson correlations between empirical and predicted values), which were subsequently averaged to obtain a metric for assessing the prediction performance of the models under the different conditions of data processing and modeling considered here.

To test for the statistical significance of our prediction results, we employed a label-shuffled permutation test (Winkler et al., 2014) within the framework of a 5-fold CV 500 times. For each iteration of the permutation test, the behavioral scores were randomly shuffled among subjects, and then a 5-fold CV procedure was conducted based on the shuffled scores. This way, chance-level prediction distributions (i.e., empirical null distributions) were generated, which were subsequently used for comparison with the observed prediction accuracies (correlations) under each data processing condition, using a significance threshold of *p* < 0.05. We also compared the prediction performance between I-EC and M-EC features, and a sample-dependent Cohen’s *d* value was calculated to assess the effect size.

As the number of features used for prediction may influence the discriminative power and model complexity of a machine-learning approach (Cai et al., 2018), we estimated the number of features that were frequently selected for model training and prediction across CV loops. This may also help to evaluate the contribution of every feature to the prediction results. During the prediction procedure, the PEB analysis was performed on each CV iteration for every subject training set, where different patterns of EC edges can be obtained. We followed such variability and calculated the relative frequencies of the features selected for predicting individual RT and age using repeated 5-fold CV. To better visualize feature contribution maps for each case, only EC parameters covarying with RT or age scores and selected by PEB with frequency ≥ 80% across all CV trials were presented. In parallel with extracting prediction contributions of EC features with the frequency of feature selection, we also estimated the average number of EC features selected by PEB across all CV iterations.

### GLM and DCM Designs

The types of event-related and block-based GLM designs (Huettel, 2012; Logothetis, 2008) were found to influence DCM parameter estimation (Zhang et al., 2024) and model selection (Daunizeau et al., 2011). Although the event-related design is currently more recommended for modeling brain activity (Petersen & Dubis, 2012), the impact that the type of GLM used in task fMRI data analysis has on the prediction results has not been clarified. Thus, we utilized a LASSO-regularized linear regression model to compare prediction correlations between GLM/DCM designs.

### CV Schemes and Predictive Models

Given the reported impact of CV schemes on prediction results in machine-learning studies (Varoquaux et al., 2017), we performed another two analyses in parallel to the repeated 5-fold CV (see the Machine-Learning Prediction Analysis section): repeated 10-fold CV and LOOCV analyses. Furthermore, a ridge-regularized linear regression model (Saunders et al., 1998) was employed as an alternative machine-learning algorithm to validate our prediction results obtained by the LASSO regression model. These parallel analyses were intended to help us evaluate the influence of CV schemes on the prediction results.

### BMR

BMR is an approach of DCM that compares evidence from different reduced models with certain combinations of coupling parameters (network edges) switched off (see the DCM Specification and Analysis section) (Friston et al., 2016). This approach can be used to identify the “best” reduced model instead of incorporating a “full” model, where all network edges are allowed to exist and will be optimized by DCM (Zeidman, Jafarian, Seghier, et al., 2019). This BMR approach has largely been applied in data-driven DCM prediction studies (Beck et al., 2021; Diersch et al., 2021), while its impact on prediction outcomes has not been well documented. To assess a possible improvement in individual predictions produced by BMR, an exhaustive search (*spm_dcm_peb_bmc.m*) was included in our prediction workflow (Figure 3c). This automatically evaluated all parameters of the full-connection model by analyzing PEB files generated from the training set. This algorithm explored different reduced models by selectively removing parameters and retaining those that contribute most significantly to model evidence (Friston et al., 2016). When a reduced model was determined, a threshold of PP > 95% was utilized to mask and extract individual EC features used for prediction.

### FC

To investigate prediction accuracies obtained from task-evoked FC, we considered both full task-evoked and task-residual FC (Zhao et al., 2023). The former type of FC was estimated by the pairwise correlation of the preprocessed task-evoked BOLD time series extracted for individual subjects from ROIs of the SRC network, which were the same as those time series specified for EC calculation by DCM. The latter type of FC was estimated by the pairwise correlation between the SRC ROI-based time series of individual subjects after regressing the timing of the experimental event sequence from the BOLD signals (Cole et al., 2014; Cole et al., 2019).

To predict individual performance or age from FC, we adapted the widely used connectome-based CPM approach (Shen et al., 2017). In particular, we applied a sparse feature selection and created two FC masks out of the top 10% of FC edges that were highly positively or negatively correlated with the prediction targets of RT or age (https://github.com/rorytboyle/flexible_cpm). These two masks were then merged into one and used for feature extraction for individual subjects. The selected individual features were then submitted to the LASSO-regularized linear regression model to predict individual age and RT according to the previously described 5-fold CV scheme. Note that, for a better comparison between prediction results for FC and EC, we did not sum up the selected individual FC features as in the default CPM approach (Shen et al., 2017). Rather, we simultaneously used all masked FC edges of individual subjects as separate features.

## RESULTS

In this study, we investigated how the task-evoked EC can be used to predict behavioral/phenotypic characteristics (RT performance and age) of individual subjects and how it could be influenced by conditions of fMRI data processing and the respective decisions that the researcher would have to make before and during the prediction analysis. We thus evaluated the influence of (a) using intrinsic (I-EC) or task-modulated (M-EC) EC as given by the DCM-derived connectivity matrices A and B, respectively (see the Methods section), (b) using event-related versus block-based GLM and DCM designs, (c) and applying BMR for DCM. Furthermore, we examined the influence of other methodological aspects of the prediction analysis. In particular, we evaluated the influence of different CV schemes and the prediction algorithm used (LASSO regularized linear regression utilized vs. ridge regression). Finally, we compared our EC-based prediction results with those obtained from using task-evoked FC as predictive features. Briefly, we observed that (a) different modalities of EC demonstrated different prediction accuracy for age and RT, where M-EC showed higher prediction accuracy for individual RT, but lower prediction accuracy for individual age than did I-EC; (b) the event-related GLM and DCM designs displayed higher accuracy in predictions than did the block-based designs; (c) employing BMR did not largely improve prediction accuracy; (d) repeated 10-fold CV and LOOCV analyses presented similar prediction patterns to that of repeated 5-fold CV, and the results obtained using LASSO were largely confirmed by using ridge regression; and (e) task-evoked FC showed higher prediction accuracy for age than did EC, but limited prediction performance for RT.

### Task-Evoked EC and Its Relation to Behavior

**Figure 4.**
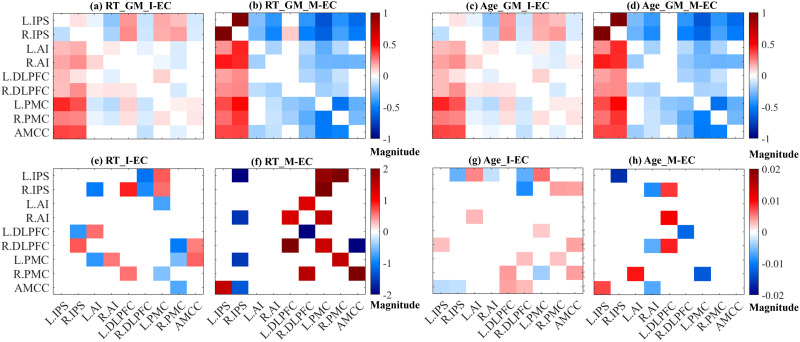
Examples of the group-mean and the behavior-related EC within the SRC task network derived from the event-related design. Panels a–d depict the group-mean EC, I-EC, and M-EC, and panels e–h depict behavior-related EC matrices A and B when either mean-centered RT or mean-centered age was used as the second column in the PEB design matrix (see the Methods section). The SRC task-related network nodes (group-level ROIs) are indicated on the axes (for abbreviations, see Figure 1). The color bars in panels a–d represent the magnitude range of the group-mean EC parameters, while the color bars in panels e–h indicate the magnitude range of behavior-related EC parameters (either for mean-centered RT or mean-centered age). These values depict the strength of the I-EC and M-EC, respectively, within the SRC task-related brain network.

Task-evoked EC investigated in this study was inferred using DCM within the SRC task-related brain network (Figure 2) for all individual subjects. The individual DCM estimations were then summarized to analyze group-level EC by the PEB technique. To illustrate the EC patterns at the group level for the whole subject sample, we calculated the I-EC and M-EC parameters (network edges) with a high PP of > 95% (Zeidman, Jafarian, Seghier, et al., 2019). The group-mean EC reflecting the connectivity averaged over all subjects and the behavior-related EC reflecting the connectivity covariance with behavioral/phenotypic scores (see the Methods section) are illustrated in Figure 4 for RT- and age-related PEB analyses. For both PEB analyses, the group-mean I-EC featured similar connectivity intensity (0.13 in Figure 4a and 0.12 in Figure 4c) as given by the absolute average of the connectivity parameters with PP > 95%. The group-mean M-EC also showed a similar connectivity intensity for both RT and age (0.33 in Figure 4b for RT and 0.34 in Figure 4d for age), which was higher overall than that of I-EC (compared with Figure 4a and Figure 4c).

Together with the group-mean EC, by the PEB approach, we also calculated the EC parameters that strongly covaried (PP > 95%) with individual RT or age (Figures 4e–4h), where we again observed a higher connectivity intensity of M-EC than I-EC (for RT: 1.9 for M-EC and 0.72 for I-EC; for age: 0.01 for M-EC and 0.004 for I-EC). In summary, M-EC parameters exhibited stronger connectivity intensities associated with both individual RT and age, as compared with I-EC parameters.

### Predicting Individual RT and Age by Task-Evoked EC

**Figure 5.**
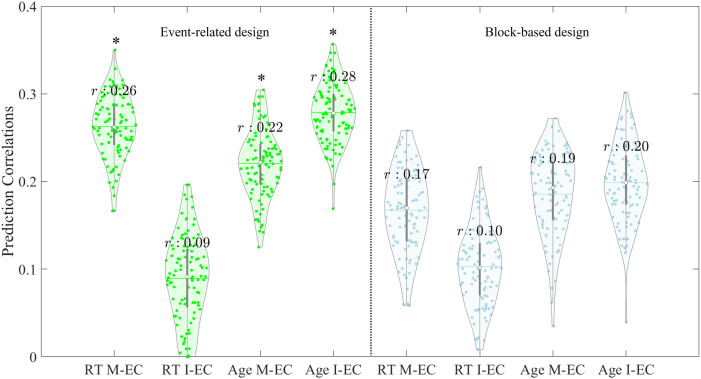
Accuracy distributions of prediction results derived from 100 repetitions of 5-fold CV using LASSO-regularized linear regression for event-related GLM designs (left panel, denoted in green) and block-based designs (right panel, denoted in blue). The correlations between observed and predicted RT and age, based on features from intrinsic effective connectivity (I-EC) and task-modulated effective connectivity (M-EC) matrices, are illustrated as violin plots, with the corresponding labels displayed on the horizontal axis. The mean correlation (*r*) is calculated by averaging across all repetitions, while each dot represents a single repetition of the prediction correlation derived from one 5-fold CV instance. Asterisks (*) indicate statistical significance in comparisons between the averaged correlations across the 100 repetitions and those from the corresponding 500-times permutation test (see Supporting Information Figure S2).

Based on the observed connectivity patterns of I-EC and M-EC (Figure 4), we evaluated how well the task-evoked EC predicts individual phenotypical and behavioral characteristics. We calculated prediction accuracy (correlation between empirical and predicted values) for the prediction of RT and age and plot the respective distributions obtained after 100 repetitions of the 5-fold CV (see the Methods section) in Figure 5 for the considered cases of the event-related and block-based GLM and DCM designs for both I-EC and M-EC (see also Supporting Information Table S1). Our results revealed that, in the case of choosing an event-related GLM and DCM design, much better performance in predicting RT was achieved with M-EC features as compared with I-EC features (mean correlation *r* = 0.26 vs. *r* = 0.09, Cohen’s *d* = 3.2; Supporting Information Table S1). On the other hand, I-EC (vs. M-EC) showed higher accuracy in predicting subjects’ age (mean *r* = 0.28 vs. *r* = 0.22, Cohen’s *d* = 1.1; Supporting Information Table S1). For the block-based GLM and DCM design, both M-EC and I-EC showed rather low prediction performance for age (mean *r* = 0.19 and *r* = 0.2, Cohen’s *d* = 0.2) and even less so for RT (mean *r* = 0.17 and *r* = 0.1, Cohen’s *d* = 1; see Supporting Information Table S1).

From the eight prediction conditions illustrated in Figure 5, we found that only three cases of the event-related GLM design appeared to be statistically significant (Figure 5, indicated by asterisks), which were evaluated based on the permutation tests (see Supporting Information Table S1 and Supporting Information Figure S2). The features of both I-EC and M-EC were predictive for subjects’ age, but only M-EC was predictive for individual RT if derived from the event-related GLM and DCM design. Interestingly, all considered conditions of the block-based design led to insignificant prediction results (Figure 5). These findings suggest that focusing on the event-related design may improve the prediction accuracy related to interindividual variability and behavioral characteristics when using task-evoked EC. Furthermore, our findings may also suggest focusing on M-EC when task-related behavioral characteristics (RT) are to be predicted, as it strongly outperformed I-EC. While both M-EC and I-EC were able to predict age to a moderate degree, the latter showed a somewhat better prediction performance.

**Figure 6.**
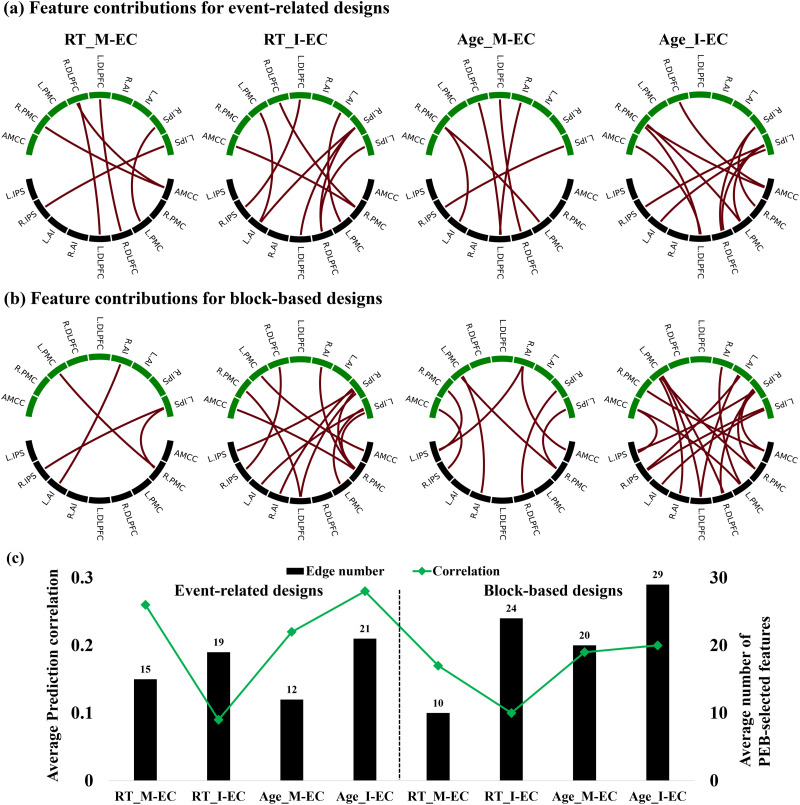
Feature contributions of specific EC edges frequently selected (panels a and b) and the average number of selected features (panel c) across all CV training loops with 100 repetitions for predicting behavioral scores. Panels a and b highlight individual EC edges selected as informative features during predictive modeling with a high frequency (≥80%) for event-related and block-based designs, showcasing the specific connections that contribute most significantly to the predictions. In panels a and b, black nodes denote “From” regions, while green nodes indicate “To” regions. The plot titles indicate the prediction target score (RT or age) and EC modality (I-EC or M-EC). In contrast, panel c presents the average number of EC features selected in each prediction loop, regardless of selection frequency, illustrating the overall relationship between prediction correlation (solid green line, left axis) and the average number of EC features (black bars, right axis) across all CV training loops. The numbers above the bars represent the average number of EC features selected by PEB analysis for model training and prediction.

We also evaluated how different features (edges of EC) were used in the prediction process. This way, we estimated how frequently one or another feature was selected for the model training and prediction and considered the frequently selected features participating with a frequency ≥ 80% across all CV loops (Figures 6a and 6b). The number of such active features highly contributing to prediction can be compared with an average number of features provided by PEB for model training and prediction at CV instances (Figure 6c).

For the event-related GLM and DCM design, we found that the I-EC versus M-EC subnetworks actively contributing to prediction were different for RT prediction including nine and six edges, respectively (Figure 6a). The corresponding average numbers of I-EC and M-EC parameters selected by PEB for RT prediction were 19 and 15, accordingly (Figure 6c). The larger number of the active features (EC parameters) did not, however, consistently positively contribute to prediction accuracy, and more RT-related features of the denser I-EC failed to significantly predict RT measured in the SRC task (Figure 6c). The discussed behavior-related SRC subnetworks also existed for the age prediction and were different from those for RT prediction as well as for both I-EC and M-EC (Figure 6a). Here, in contrast, we observed a slight trend that the larger number of EC parameters selected for the age prediction positively contributed to prediction quality. Notably, the features of M-EC resulted in a somewhat weaker age prediction accuracy than those of I-EC (Figures 6a and 6c).

For the block-based GLM and DCM design, similar observations can be reported, where very different subnetworks of the SRC network actively participated in RT and age prediction by I-EC and M-EC. In particular, only 4 and 12 edges were frequently selected for RT prediction out of 10 and 24 features that on average were suggested by PEB from all M-EC and I-EC connections, respectively (Figures 6b and 6c). Again, a smaller M-EC subnetwork can lead to somewhat better RT prediction than the larger I-EC subnetwork. The EC connectivity patterns frequently employed for prediction are also very different between the two considered GLM and DCM designs of the event-related and block-based types.

In summary, the stability, density, and connectivity pattern of the behavior-related SRC subnetwork frequently selected for model training and prediction could not immediately be assigned to the observed prediction accuracy and might need additional investigation. Nevertheless, by such a 5-fold CV subsampling, we found EC connections of the SRC network that robustly covaried with the considered behavioral scores and can be used for their investigation.

### Predicting RT and Age From Task-Evoked FC

We also applied our approach to the prediction of RT and age based on task-evoked FC features calculated by Pearson correlations between task-evoked BOLD time series extracted from the SRC-related network nodes. Despite methodological differences between the EC- and FC-based approaches, we found that the two types of FC (full task-evoked and task-residual; see the Methods section) performed relatively well in the prediction of individual age (*r* = 0.29 ~ 0.31). However, FC succeeded much less in predicting RT for both GLM designs (*r* = 0.07 ~ 0.13), where the task-evoked EC, especially M-EC for the event-related GLM-DCM design, outperformed the task-related FC (see Figure 5 and compare Table 1 and Supporting Information Table S1).

**Table 1.** Performance of functional connectivity (FC) features in predicting individual reaction time (RT) and age.

GLM design	Event		Block	
	full	res	full	res
RT	0.11 ± 0.04	0.13 ± 0.04	0.07 ± 0.04	0.10 ± 0.05
Age	0.29 ± 0.03	0.30 ± 0.04	0.31 ± 0.04	0.31 ± 0.04

Individual RT and age were separately predicted by individual FC using CPM implemented in a 5-fold CV scheme and repeating the predictive model for each target 100 times to derive an averaged correlation between empirical and predicted values. Considering empty edges in feature selection procedures, we used a sparsity function that selected the top 10% positively and negatively target-correlated edges, respectively, rather than a fixed *p* value for thresholding, in every training set for modeling.

### CV Analysis

**Figure 7.**
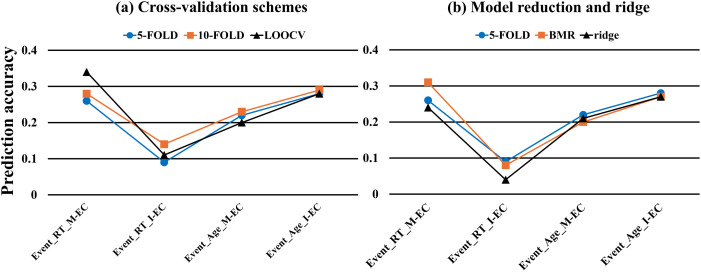
Overview of the mean prediction accuracy of all considered conditions in predicting individual RT and age under the event-related (Event) GLM design with features selected by behavior-related PEB analyses from EC modalities of M-EC and I-EC indicated on the horizontal axis. Plots (a) and (b) indicated the conditions involving cross-validation schemes including 5-fold CV, 10-fold CV, and LOOCV, and applications of the BMR and ridge-regularized regression. Except for LOOCV, the prediction accuracies of all conditions were represented by averaged correlations between empirical and predicted behavioral scores from the repeated *k*-fold cross-validation analyses.

We calculated prediction accuracies for different CV schemes for the event-related GLM-DCM design, including a repeated 10-fold CV (Supporting Information Table S2 and Supporting Information Figure S3) and LOOCV (Figure 7a and Supporting Information Table S3). The 10-fold CV revealed a pattern similar to the 5-fold CV, where we still observed that M-EC predicted RT much better than did I-EC (mean *r* = 0.28 vs. *r* = 0.14). On the other hand, I-EC manifested a somewhat better correlation in predicting individual age than did M-EC (mean *r* = 0.29 vs. *r* = 0.23).

For the LOOCV, we also found a similar pattern of prediction results, where M-EC presented a much better correlation than I-EC in predicting individual RT (mean *r* = 0.34 vs. *r* = 0.11) but lower prediction accuracy for age (mean *r* = 0.2 vs. *r* = 0.28).

Despite the prediction correlations were somewhat varying among different CV schemes, we nevertheless observed robust prediction results related to M-EC and I-EC across different CV schemes (see Figure 7a). Indeed, M-EC much outperformed I-EC in predicting RT for all cases considered. EC connectivity may also be used for age prediction, where I-EC demonstrated somewhat better prediction accuracy than did M-EC.

### Model Reduction Analysis

We also examined the case when the full DCM model employed above was replaced by a reduced model based on the BMR approach. The prediction performances of I-EC and M-EC for individual RT and age using BMR-extracted features (PP > 95%) are displayed in Figure 7b, Supporting Information Figure S4, and Supporting Information Table S4. The results obtained were similar to our previous observations for the full DCM model. In particular, as before, M-EC showed a much higher prediction accuracy (correlation) for RT than did I-EC (*r* = 0.31 vs. *r* = 0.08), and I-EC showed a stronger prediction correlation for age than did M-EC (*r* = 0.27 vs. *r* = 0.2). These findings indicate that considering the DCM-reduced models via BMR hardly influenced the prediction results and conclusions derived for the full model (see Figure 7b).

### Predictive Models

The prediction analysis using ridge-regularized linear regression in a 5-fold CV scheme yielded similar results (Figure 7b and Supporting Information Table S5), which validated our LASSO-regularized prediction model. For the event-related cases, M-EC (vs. I-EC) showed higher prediction accuracy for RT (*r* = 0.24 and *r* = 0.04), but lower accuracy for age (*r* = 0.21 and *r* = 0.27).

## DISCUSSION

In this study, we investigated and compared the prediction performance of intrinsic and task-modulatory EC patterns obtained from DCM of task fMRI data. For this, we explored machine-learning-based predictions of individual age and RT performance by LASSO- and ridge-regularized linear regression using two types of task-evoked EC: I-EC as given by matrix A of the DCM model and task-modulatory EC (M-EC, matrix B). We adopted a CV-based PEB analytical strategy to extract I-EC or M-EC parameters as predictive features to avoid data leakage in predicting individual behavioral or phenotypical scores. We compared the prediction results for two different GLM and DCM designs of task fMRI processing and EC estimation, the event-related and block-based designs. We also calculated and compared the prediction results for different CV schemes, when BMR was applied to DCM, as well as when using ridge regression as an alternative machine-learning model and task-evoked FC patterns as feature space.

Our results demonstrated that (a) the event-related GLM-DCM design performed better at predicting individual phenotypes than did the block-based GLM design; (b) using M-EC led to a higher prediction accuracy (correlation) for RT prediction, while I-EC was better for the age prediction in the case of an event-related design; (c) employing BMR did not largely affect prediction accuracy; (d) different CV schemes showed similar prediction patterns, where LOOCV was showing more optimistic results than the 5-fold CV scheme in some cases; (e) the results obtained for the LASSO-based predictive model were largely confirmed by models using ridge regression; (f) task-evoked FC (vs. EC) showed a higher prediction accuracy for age, but a lower accuracy for RT, where the event-related M-EC outperformed FC.

### SRC Network

The SRC task is a well-established experimental paradigm to study cognitive action control during conflict processing by employing measures such as RT and error rates in response to task stimuli (Fitts & Deininger, 1954), where shorter RT and higher accuracy is typically observed in a congruent condition, as compared with the incongruent one. In contrast to RT, previous studies also reported an age-related difference in both behavioral performance and connectivity among brain regions (Langner et al., 2015; Proctor et al., 2005). In line with these studies, our findings demonstrated that both age and RT can be predicted by brain EC, albeit the prediction accuracy ranged from weak to moderate (see Figure 5). Moreover, our findings revealed distinct EC patterns that contributed to predicting individual RT and age, as indicated by feature contributions of EC (Figures 6a and 6b). Although we selected nodes within the SRC network, where the incompatibility effects were present, the connections among these regions exhibited varying degrees of contribution to age and RT prediction. The dissociation of the entire SRC network into different subnetworks actively contributing to age and RT prediction could reveal differently weighted roles of the network nodes (brain regions) and their connectivity for various prediction targets. This could help to evaluate potentially distinct associations to, for example, individual age and RT within the SRC network.

### I-EC and M-EC

To our knowledge, the differential roles underlying the prediction performance of I-EC and M-EC extracted from task-evoked fMRI have not been previously investigated. Although I-EC is derived from task fMRI data, it shares similarities with intrinsic connectivity, as I-EC is mathematically freed from task-related variance, allowing it to reflect condition-invariant, spontaneous baseline activity during task-evoked brain states (Marreiros et al., 2008; Zeidman, Jafarian, Corbin, et al., 2019). However, it is important to note that I-EC retains some influences from the task context, making it an imperfect analogy to resting-state data. Despite this shortcoming, I-EC still captures intrinsic connectivity dynamics akin to resting-state connectivity, making it a useful tool for studying intrinsic connectivity even in task-evoked data.

For the event-related GLM design, we found that M-EC exhibited a higher prediction correlation for individual RT than did I-EC. This finding is comparable with the literature on the predictiveness of task-evoked versus resting-state FC, which suggests that task-evoked connectivity may better capture task-specific behavioral differences and variability than does resting-state connectivity and thereby lead to superior prediction results (Gbadeyan et al., 2022; Zhao et al., 2023). This advantage may stem from task manipulations that accentuate brain functional correlation patterns relevant to behavior (Greene et al., 2018; Zhao et al., 2023). Compared with resting-state connectivity, the specificity of a given task, constrained nature, and reduced variability of task-evoked connectivity may allow for better capturing some individual behavioral differences (Buckner et al., 2013; Elton & Gao, 2015; Finn et al., 2017; Geerligs et al., 2015).

We found that I-EC (vs. M-EC) exhibited a slightly higher accuracy in predicting individual age in the event-related GLM design. This is a different pattern of prediction based on I-EC versus M-EC as we observed for RT prediction in spite of some moderate correlation between RT and age. Both types of EC have been identified as decent predictors of individual age (Diersch et al., 2021; Tsvetanov et al., 2016). For instance, Tsvetanov et al. (2016) predicted individual age (*r* > 0.3) using resting-state EC parameters estimated within and between the brain’s main networks. Similarly, Diersch et al. (2021) demonstrated that hippocampal excitability of the task-evoked EC was influenced by aging (*r* = 0.29), showing the capability to classify participants into younger and older groups. These findings suggest that both I-EC and M-EC can be influenced by individual age, which justifies their consideration as features for age prediction. The advance of resting-state connectivity for age prediction also aligns with previous studies (Ferreira & Busatto, 2013; Millar et al., 2022; Xiong et al., 2023). For instance, evidence from a multimodal MRI study for brain age prediction (Xiong et al., 2023) has shown a modality-specific prediction difference between the resting-state and task-evoked datasets, where the former outperformed the latter. This difference may relate to the dissociation of BOLD variability between them, where the resting-state BOLD variability may capture different sources of variance and be more sensitive to global age-related (e.g., vascular and white matter) factors than is task-evoked variability (Millar et al., 2020). In line with previous literature, our findings suggest that brain I-EC and M-EC may contribute differently to predicting individual RT and age. Although we noticed that M-EC and I-EC components in the block-based designs failed to predict individual RT and age (*p* > 0.05), the observed patterns suggest a potential tendency between M-EC and I-EC components in their predictive performance for age and RT.

### GLM and DCM Designs

Despite minor differences, the LOOCV and *k-*fold CV prediction results have shown higher prediction correlations for the event-related design compared with the block-based design (Figure 5). Both I-EC and M-EC estimated from the block-based design resulted in lower and insignificant prediction correlations with individual RT and age, as compared with EC estimated for the event-related design. Previous studies (Bühler et al., 2008; Tie et al., 2009) have compared the impact of GLM designs on task-evoked activations and reported that the block-based design resulted in more widespread brain activations that were not specifically involved in the task compared with the event-related design (Bühler et al., 2008). This difference may be attributed to different hemodynamic response shapes modeled by GLM designs (Mechelli, Henson, et al., 2003; Mechelli, Price, et al., 2003). The event-related design was found to explain the signal variance better, with predicted hemodynamic responses peaking earlier and returning to the baseline later (Mechelli, Henson, et al., 2003). In addition, our previous study (Zhang et al., 2024) reported an overall higher M-EC strength for the event-related design, relative to the block-based design. Taking these results together, the higher prediction correlations with individual performance in the event-related GLM-DCM design may be related to its stronger sensitivity to individual task events. This makes the event-related design a promising modeling approach for better prediction and understanding of behavioral characteristics related to the tasks, for example, RT, as in this study.

### CV Schemes and Machine-learning Algorithms

The present study employed a CV-based PEB analytical strategy, which enabled us to estimate behavior-modulated EC efficiently and avoided possible data leakage in prediction. In particular, extracting EC edges with PP > 95% as features from the entire sample, instead of extracting them from every CV loop for the training set, may lead to overoptimistic prediction results (Mwangi et al., 2014). Additionally, despite the instability and bias reported for LOOCV (Varoquaux et al., 2017), our *k-*fold CV scheme (repeated 100 times) has still displayed a notably consistent prediction pattern also with LOOCV (Figure 7a). In some cases, we, however, observed an enhanced prediction correlation for LOOCV, relative to the 5-fold CV scheme, for example, for M-EC in RT prediction (Supporting Information Tables S1 and S3). Furthermore, the ridge-regularized machine-learning analysis confirmed our results obtained from the LASSO-regularized analysis (Supporting Information Table S5). The relatively consistent prediction patterns obtained across various CV schemes and machine-learning algorithms support the robustness and stability of our findings.

### BMR

We explored the possible benefits of employing BMR of DCM for predicting individual RT and age with both types of EC (Figure 7b). However, we observed that the impact of BMR on prediction results was relatively small (Supporting Information Figure S4). BMR refers to the procedure of comparing different reduced models initiated from a prespecified model using Bayesian inversion to find a “winning” model that fits the neural data best (Zeidman, Jafarian, Seghier, et al., 2019). This procedure iteratively switches off the connectivity parameters and compares the PP of these reduced models until the “best” model is captured (Friston et al., 2016). Several studies have applied BMR to remove connectivity parameters from the full model and to predict individual differences based on the EC of the reduced model (Beck et al., 2021; Diersch et al., 2021). However, the impact of BMR on prediction results has remained unclear without direct comparisons. In our study, we did observe a slightly higher prediction accuracy with BMR in the case of RT prediction using M-EC features (Figure 7b). However, there was still a notably consistent prediction pattern across the cases with BMR and those using the full DCM model. The current findings thus indicate that the impact of BMR on the prediction results is relatively small. More detailed and comprehensive investigations are needed to evaluate the role and significance of BMR in DCM-based prediction analyses.

### Joint EC Analysis of I-EC and M-EC

Considering that both I-EC and M-EC components can contribute to changes in neuronal states, we conducted additional prediction analyses with combined features including both I-EC and M-EC components together, and compared the results with those using each component separately (Supporting Information Figure S5). In the event-related design, the combined EC model predicted age with a mean correlation of 0.28 and RT with 0.26. For the block design, age prediction had a correlation of 0.20, while for RT, it was 0.15. Across different CV schemes, the combined analyses showed prediction correlations ranging from 0.28 to 0.33 for RT and from 0.29 to 0.31 for age. When using BMR and ridge-regularized linear regression, the BMR model achieved a prediction correlation of 0.29 for both RT and age, whereas the ridge regression model showed a prediction of 0.24 for RT and 0.28 for age. These results were not significantly different from predictions using I-EC or M-EC alone, indicating that isolating I-EC and M-EC provides comparable prediction accuracy. Additionally, this approach offers more specific insights into which aspect of task-evoked EC is involved in the respective brain-behavior relationships.

### FC

We examined task-evoked FC and compared its prediction performance with task-evoked EC. Our findings revealed that task-evoked FC was more predictive of age than was EC, whereas it was less predictive of RT than was task-evoked M-EC (see Table 1 and Supporting Information Table S1). Consistent with previous literature (Greene et al., 2018; Tsvetanov et al., 2016; Zhao et al., 2023), which demonstrated that FC could predict individual task performance and age with a correlation generally greater than 0.2, our results show a comparable prediction of age and RT using task-evoked FC from the SRC network, although RT prediction was lower. Furthermore, although EC was thought to be more discriminative than FC in identifying brain “fingerprints” from a previous resting-state fMRI study (Geng et al., 2018), the moderate improvement in age prediction by task-evoked FC highlights a sophisticated role of task-evoked connectivity measures. This may suggest that FC provides valuable insights in task-evoked contexts, especially for age prediction. Of note, we should also consider methodological variations in the prediction pipeline between EC- and FC-based predictions. These methodological differences in feature selection methods and selected feature numbers might be another source of the observed differences in prediction performance.

### Limitations

Some limitations of our approach should be considered and possibly addressed in future research. First of all, as in other task-related studies, also in this study, we extracted group-based ROIs of brain activation based on the entire sample, which may be subject to the risk of data leakage in subsequent prediction analysis. The possible data leakage problem might be due to the whole-sample second-level fMRI analyses applied to training and test sets together instead of performing them separately for the given training set in each CV loop (Kapoor & Narayanan, 2023; Rosenblatt et al., 2024). The impact of this problem on prediction results, however, is not immediately evident and has to be investigated in a dedicated separate study, which is computationally expensive and would require large computational resources. Here, we nevertheless verified that the second-level fMRI analysis applied to the training sets within CV loops did not lead to notably different ROIs, as compared with the whole-sample case (Supporting Information Figure S6). It is therefore reasonable to assume that the discussed potential data leakage did not have any notable impact on our prediction results and the reported conclusions. Second, while I-EC might approximate resting-state connectivity to a large extent by removing task-related variance, it still retains some influences from the task context, which makes it an imperfect analogy to resting-state connectivity. This partial similarity may affect the comparability of I-EC and resting-state connectivity in capturing spontaneous brain activity. Future research could benefit from estimating I-EC directly from resting-state data and comparing its predictive performance with task-related M-EC to better understand their relative effectiveness and implications for behavioral prediction. Third, we employed a LASSO-regularized linear regression model, which might face problems in case of high collinearity in the data and cannot well account for (non)linear relations between the features of brain EC used for the prediction of behavioral characteristics. Although we confirmed our results using ridge regression, additional investigations are warranted to better clarify this limitation. Finally, while our study provides interesting insights into task-evoked EC within the SRC-related brain network, it is limited by its focus on a specific task paradigm, and a possible generalization of the reported results to other tasks or even to the whole-brain connectome is important. Future research could address these limitations to offer a more comprehensive understanding of brain dynamics and connectivity across different cognitive states and their relations to behavior.

### Conclusion

Our study aimed to predict individual RT and age from I-EC and M-EC and investigated the impact of a variety of data processing conditions, including types of DCM-GLM designs, application of BMR as well as diverse CV schemes. Our findings suggest that I-EC and M-EC may capture different phenotypic attributes, in spite of relatively low prediction accuracies observed for both task-evoked EC and FC regarding the prediction of RT or age. Here, M-EC demonstrated higher prediction accuracy for individual RT, whereas I-EC was better predictive of individual age. Furthermore, task-evoked EC outperformed FC in predicting individual RT but presented a slightly lower accuracy for individual age. Notably, prediction performance was significantly affected by the choice of GLM-DCM design, but only slightly influenced by other considered conditions of data processing and analysis, and using the event-related GLM-DCM design may improve prediction accuracy of task-evoked EC for individual RT and age. The presented results can contribute to better applicability of effective brain connectivity to the investigation of interindividual variability in brain-behavior relationships.

## ACKNOWLEDGMENTS

The authors gratefully acknowledge computing time on the supercomputer JURECA (Jülich Supercomputing Centre, 2021) at Forschungszentrum Jülich under grant no. “cjinm71.” We would like to acknowledge the 1000BRAINS project (Caspers et al., 2014) for providing the dataset. We are also grateful to the “ML hours” initiative from INM-7 and Kevin Wischnewski for informative and useful discussions.

## SUPPORTING INFORMATION

Supporting information for this article is available at https://doi.org/10.1162/netn_a_00447.

## AUTHOR CONTRIBUTIONS

Oleksandr Popovych: Conceptualization; Funding acquisition; Investigation; Methodology; Project administration; Resources; Software; Supervision; Writing – review & editing. Shufei Zhang: Data curation; Formal analysis; Investigation; Methodology; Resources; Software; Validation; Visualization; Writing – original draft; Writing – review & editing. Kyesam Jung: Formal analysis; Investigation; Methodology; Writing – review & editing. Robert Langner: Formal analysis; Methodology; Project administration; Writing – review & editing. Esther Florin: Conceptualization; Project administration; Supervision; Writing – review & editing. Simon B. Eickhoff: Funding acquisition; Project administration; Supervision; Writing – review & editing.

## FUNDING INFORMATION

Oleksandr Popovych, Helmholtz Association and the European Union’s Horizon 2020 Research and Innovation Programme, Award ID: 945539, 826421. Oleksandr Popovych, Deutsche Forschungsgemeinschaft (https://dx.doi.org/10.13039/501100001659), Award ID: 491111487.

## DATA AND CODE AVAILABILITY

The raw data of the 1000BRAINS project used in this study are not immediately available for public sharing because the authors do not have permission to share data. The codes are available at the (https://github.com/CogPsycho2023/DCMs2).

## Supplementary Material



## TECHNICAL TERMS

Functional connectivity (FC): Statistical correlations between brain regions' activity, indicating their functional relationships.
Dynamic causal modeling (DCM): A computational approach for inferring directed interactions between brain regions from neuroimaging data.
Effective connectivity (EC): The influence one brain region exerts over another, estimated using causal models like DCM.
Intrinsic effective connectivity (I-EC): EC reflecting baseline interactions in the absence of task-related modulations.
Task-modulated effective connectivity (M-EC): EC connections driven by specific task conditions.
Cross-validation (CV): A method for evaluating model performance by partitioning data into training and testing subsets.
General linear model (GLM): A statistical framework for modeling and testing linear relationships in neuroimaging data.
Bayesian model reduction (BMR): A Bayesian inference technique for optimizing model evidence by simplifying parameters.
Lasso: A regression technique using L1 regularization for feature selection and prediction accuracy.
Ridge regression: A regression method employing L2 regularization at the estimation of model parameters.
