# Supplementary material for: Predicting response speed and age from task-evoked effective connectivity
Source: Netw Neurosci. 2025 Apr 30;9(2):591–614. doi: 10.1162/netn_a_00447 (PMC12140579; doi:10.1162/netn_a_00447)
Supplement: Supplementary file 1 [file netn-9-2-591-s001.pdf]

Supplementary materials

for the manuscript entitled

**Predicting response speed and age from task-evoked effective connectivity**

by

Shufei Zhang[1,2], Kyesam Jung[1,2], Robert Langner[1,2], Esther Florin[3], Simon B. Eickhoff[1,2], Oleksandr V. Popovych[1,2]

[1]Institute of Neuroscience and Medicine, Brain and Behaviour (INM-7), Research Centre Jülich, Germany

[2]Institute for Systems Neuroscience, Medical Faculty, Heinrich-Heine University Düsseldorf, Germany

[3]Institute of Clinical Neuroscience and Medical Psychology, Medical Faculty, Heinrich-Heine University Düsseldorf, Germany

*Corresponding author: o.popovych@fz-juelich.de

**Supplementary Figures**

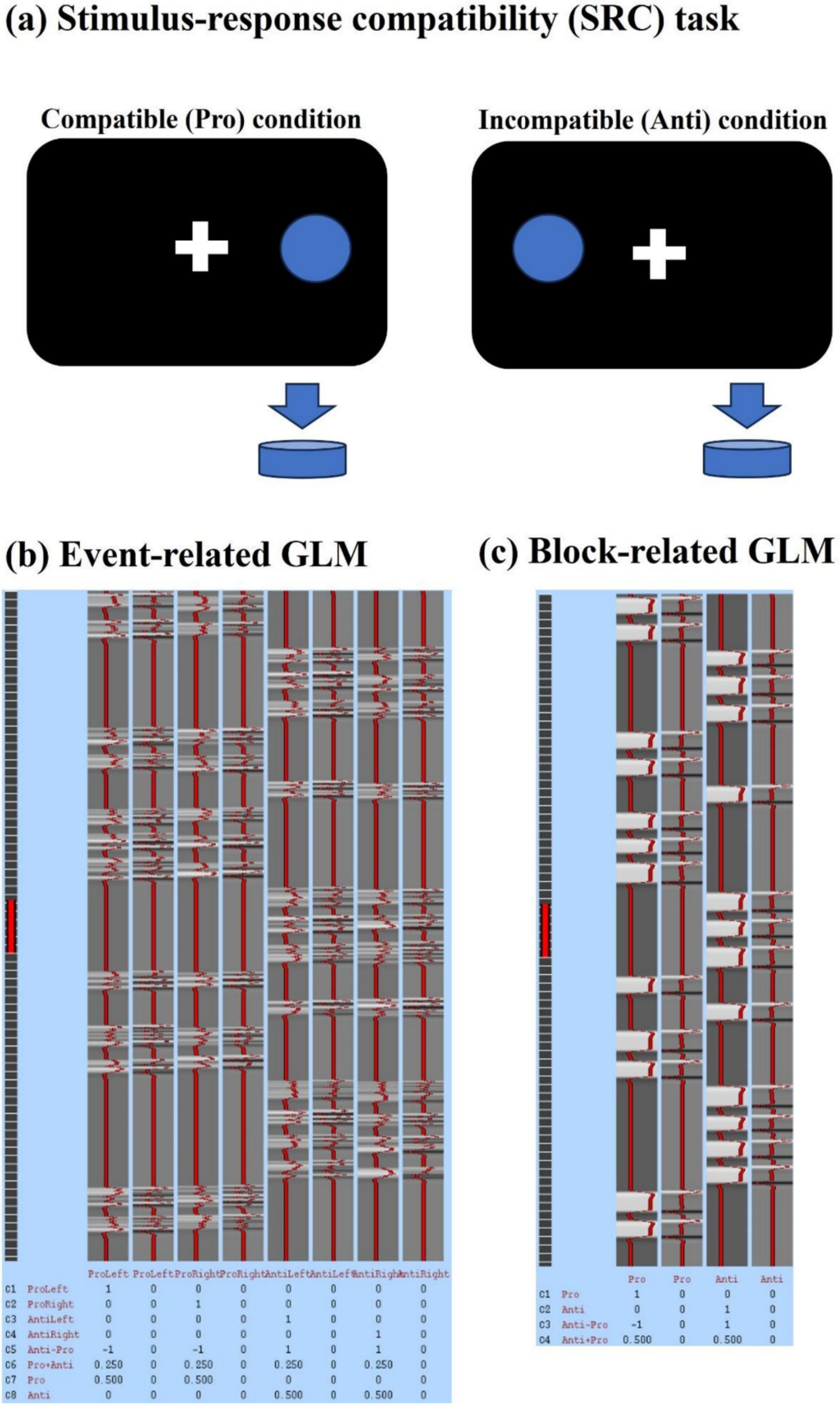


Fig. S1 The overview of the stimulus-response compatibility (SRC) task and general linear model (GLM) designs for event-related and block-based designs. (a) The SRC task includes two conditions: compatible (Pro) and incompatible (Anti) conditions. During the task, participants were instructed to respond to lateralized visual stimuli by accurately and rapidly pressing an ipsilateral (Pro) or contralateral button (Anti), respectively. (b/c) The event-related and block-based GLM designs were implemented in the task-evoked first-level fMRI

analyses, where double-gamma and temporal derivatives were utilized to model individual BOLD signals.

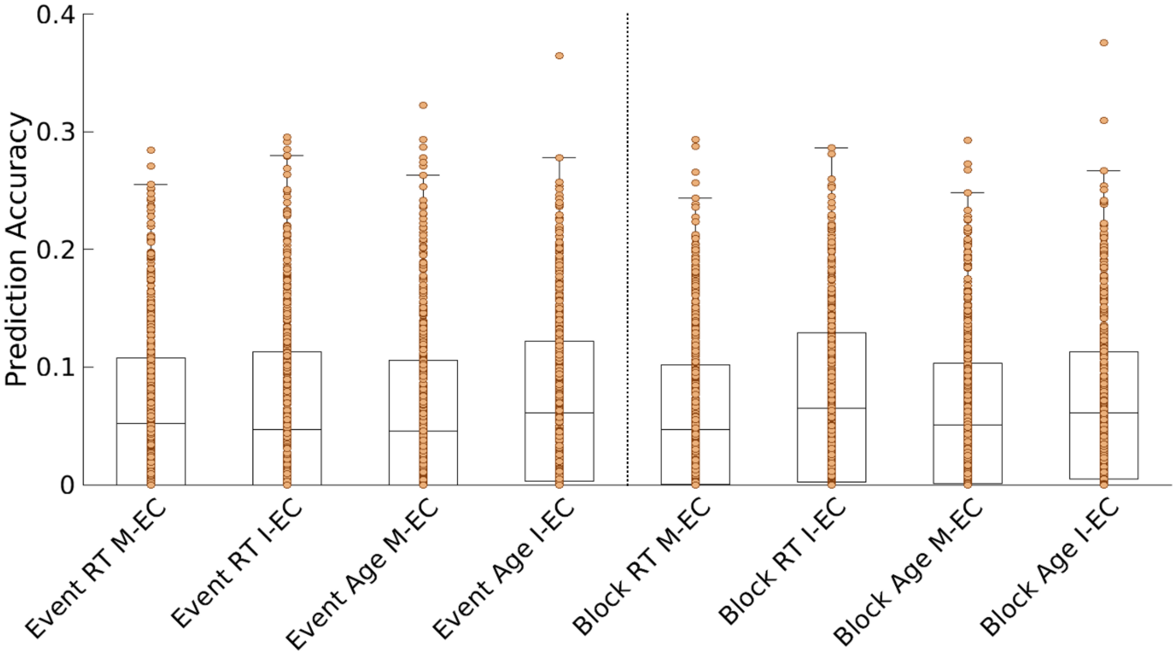


Fig. S2. A summary of label-shuffled permutation tests (500 times) obtained from block/event-related designs and intrinsic/task-modulated effective connectivity (I-EC/M-EC) for reaction time (RT) and age. The orange dot in the figure represents the prediction correlation derived from one fixed 5-fold cross-validation splitting scheme where the behavioral labels were shuffled randomly. Note, that the fixed-5-fold cross-validation splitting scheme was the one showing the highest prediction correlation in the repeating 5-fold analysis.

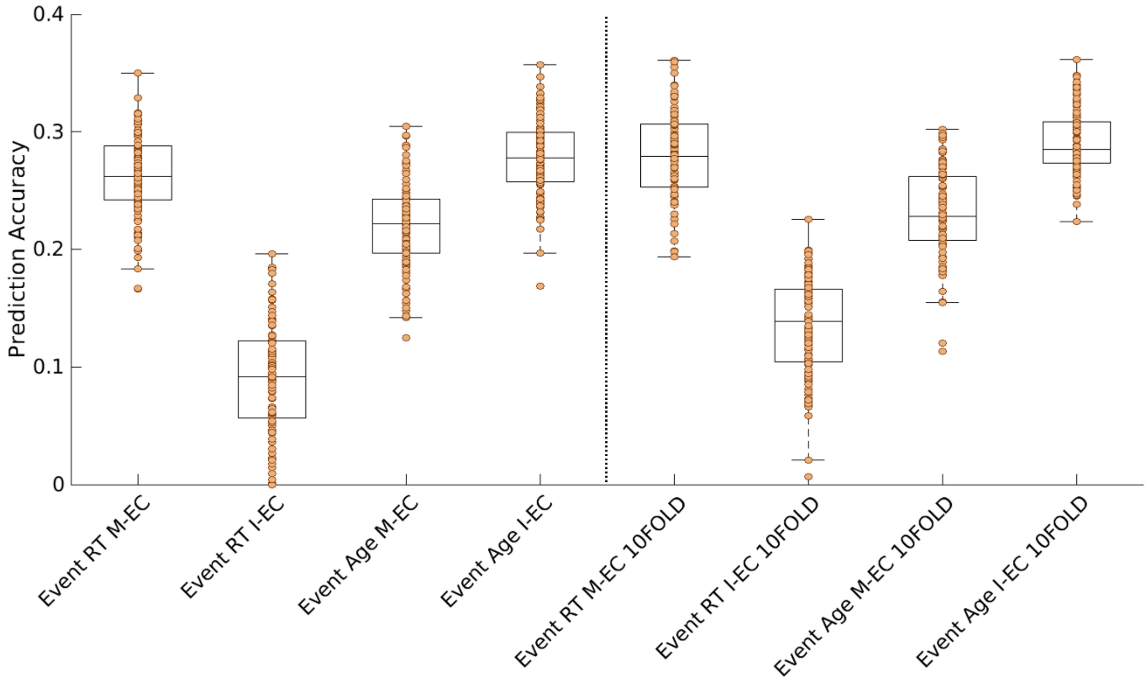


Fig. S3. A summary of prediction results obtained from the event-related design and intrinsic/modulatory effective connectivity (I-EC/M-EC) in predicting individual reaction time (RT) and age using a 5-fold (the left penal) and10-fold (the right penal) cross-validation approach. The orange dot in the figure represents the prediction correlations derived from one randomly sampled cross-validation instance.

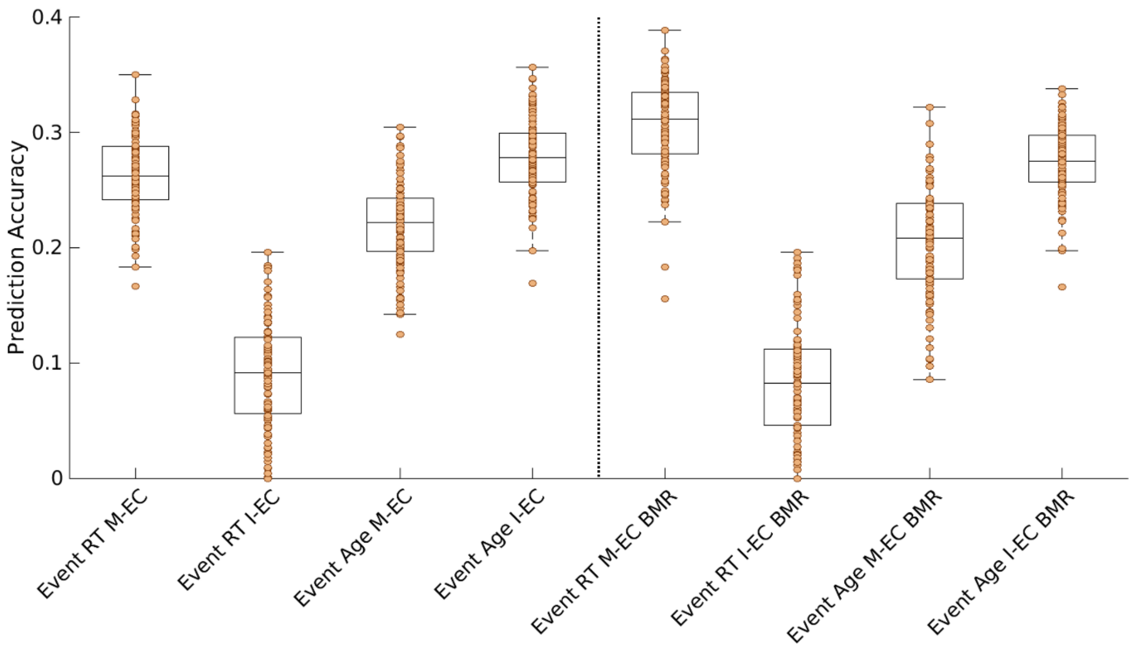


Fig. S4. A summary of prediction results obtained from the event-related design and intrinsic/modulatory effective connectivity (I-EC/M-EC) incorporating prediction cases without (the left panel) and those with Bayesian model reduction (BMR) procedure (the right penal) in predicting individual reaction time (RT) and age using a 5-fold cross-validation approach. The orange dot in the figure represents the prediction correlations derived from one randomly sampled 5-fold cross-validation instance.

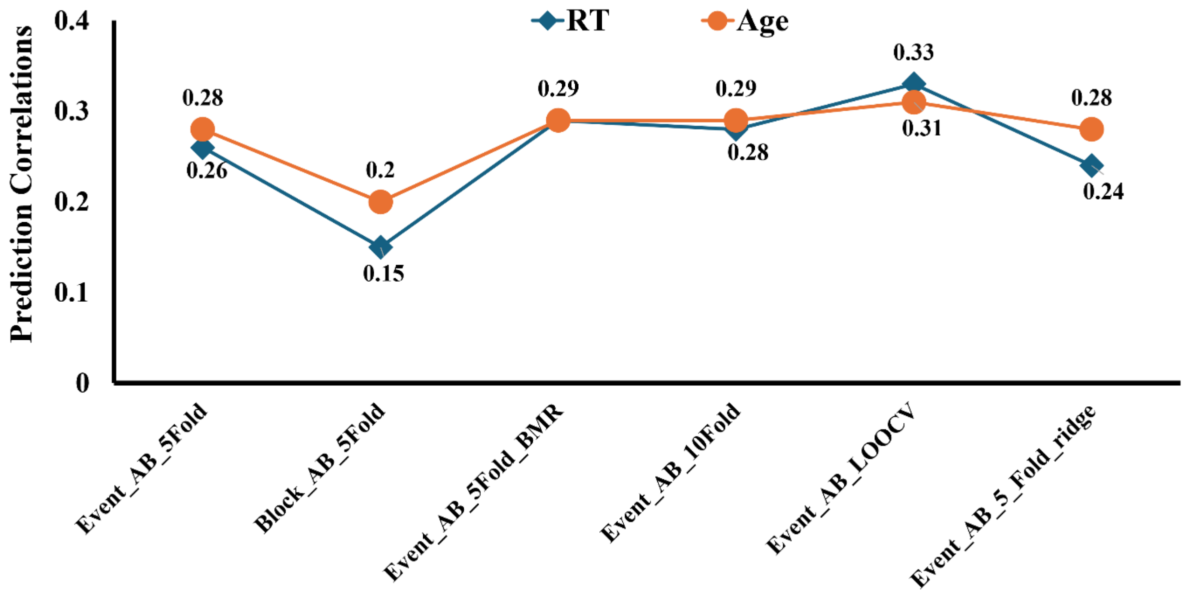

Fig. S5 Overview of the mean prediction accuracy (Pearson's *r*) across all conditions for predicting individual reaction time (RT) and age in various prediction scenarios. Each model combines intrinsic and task-modulated effective connectivity (EC) parameters, with prediction correlations calculated between empirical and predicted values. The correlations were averaged across 100 iterations of random subject splits for cross-validation (CV) analyses, except for the leave-one-out CV (LOOCV) cases. The horizontal axis represents different prediction conditions involving CV schemes including 5-fold, 10-fold, and LOOCV, applications of Bayesian model reduction (BMR), and ridge-regularized regression. The vertical axis shows the prediction correlations (*r*).

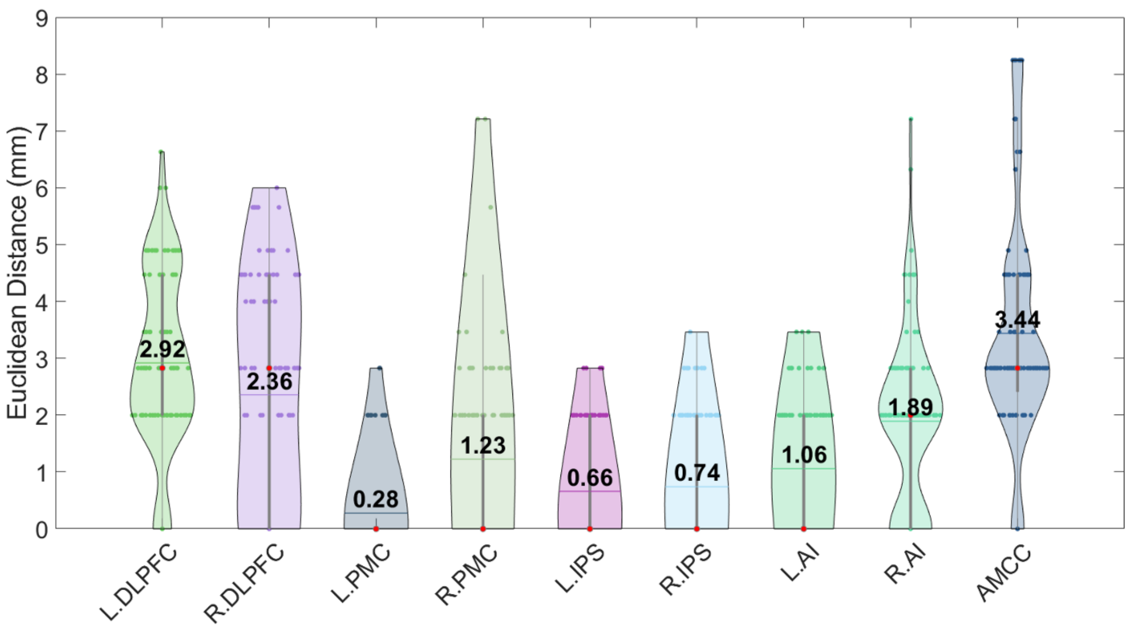


Fig. S6 Variations in Group-Level Peak Coordinates of Regions of Interest (ROIs) During 5-Fold Cross-Validation. The entire sample was split into training and testing sets using a 5-fold cross-validation (CV) approach. For each fold, second-level fMRI analyses were conducted on the training set, and ROIs were identified based on the peak coordinates derived from these analyses. The Euclidean distance between the peak coordinates of ROIs selected from each fold and the ROIs obtained from the full sample was calculated. This process was repeated 20 times, resulting in 100 points per network node. These differences

were visualized as violin plots to illustrate the distribution of variations in ROI coordinates, with the mean value for each violin shown.

**Supplementary Tables**

Table 1. Prediction performance of the features extracted from intrinsic EC (I-EC) and task-modulated EC (M-EC) for individual reaction time (RT) and age obtained by LASSO regression with 5-fold cross-validation

| Predictors | Event | | Block | |
|---|---|---|---|---|
| | M-EC | I-EC | M-EC | I-EC |
| RT | $r = 0.26 \pm 0.04$ * | $r = 0.09 \pm 0.05$ | $r = 0.17 \pm 0.05$ | $r = 0.10 \pm 0.04$ |
| Age | $r = 0.22 \pm 0.04$ * | $r = 0.28 \pm 0.03$ * | $r = 0.19 \pm 0.05$ | $r = 0.20 \pm 0.04$ |

The prediction correlations were estimated from a 100-time 5-fold CV, which employed the LASSO-regularized linear regression model. The upper number in the table cells indicates the

averaged correlation *r* and its standard deviation across repetitions. Asterisks (*) indicate a statistically significant difference ($p < 0.05$) between averaged prediction correlations and label-shuffled permutation tests (500 times), which are displayed in Supplementary Fig. S2.

Table 2. 10-fold prediction performances of M-EC and I-EC in individual RT and age

| Predictors | Event | |
|---|---|---|
| | M-EC | I-EC |
| RT | 0.28 ± 0.04 | 0.14 ± 0.04 |
| Age | 0.23 ± 0.04 | 0.29 ± 0.03 |

The prediction correlations were estimated from a 100-time 10-fold CV, which employed the Lasso-regularized linear regression model. The number in the element shows the averaged correlation between empirical and predicted values, and the second one indicates the standard deviation. M-EC: task-modulated effective connectivity; I-EC: intrinsic effective connectivity; RT: reaction time.

Table 3. LOOCV prediction performances of M-EC and I-EC in individual RT and age

| Predictors | Event | |
|---|---|---|
| | M-EC | I-EC |
| RT | 0.34 | 0.11 |
| Age | 0.20 | 0.28 |

The prediction correlations were estimated from a Leave-one-out cross-validation (LOOCV) scheme, which employed the Lasso-regularized linear regression model. The number in the elements represents the prediction correlation between empirical and predicted values. M-EC: task-modulated effective connectivity; I-EC: intrinsic effective connectivity; RT: reaction time.

Table 4. Prediction performances of M-EC and I-EC in individual RT and age with reduced models

| Predictors | Event | |
|---|---|---|
| | M-EC | I-EC |
| RT | 0.31 ± 0.04 | 0.08 ± 0.05 |
| Age | 0.20 ± 0.05 | 0.27 ± 0.03 |

The prediction correlations were estimated from a 100-time 5-fold CV, which extracted EC connections from reduced models (Bayesian model reduction) and employed the Lasso-regularized linear regression model for prediction. The number in the element shows the averaged correlation between empirical and predicted values, and the second one indicates the standard deviation. M-EC: task-modulated effective connectivity; I-EC: intrinsic effective connectivity; RT: reaction time.

Table 5. The 5-fold prediction performances of M-EC and I-EC in individual RT and age using ridge-regularized linear regression

| Predictors | Event | |
|---|---|---|
| | M-EC | I-EC |
| RT | 0.24 ± 0.04 | 0.04 ± 0.05 |
| Age | 0.21 ± 0.04 | 0.27 ± 0.03 |

The prediction correlations were estimated from a 100-time 5-fold CV, which employed the ridge-regularized linear regression model. The number in the element shows the averaged correlation between empirical and predicted values, and the second one indicates the standard deviation. M-EC: task-modulated effective connectivity; I-EC: intrinsic effective connectivity; RT: reaction time.
